# Highlighting the ‘public ‘in digital public health – a critical reflection

**DOI:** 10.1093/eurpub/ckac129.148

**Published:** 2022-10-25

**Authors:** H Zeeb, B Schüz, I Pigeot

**Affiliations:** 1 BIPS, Bremen, Germany; 2 Leibniz ScienceCampus Digital Public Health, Bremen, Germany; 3 Institute of Public Health and Nursing Research, University of Bremen, Bremen, Germany; 4 EUPHA-DH

## Abstract

What is the appropriate differentiation of digital public health versus digital health - or is there none? This is an essential question when pondering digitalisation and public health, especially with a view to the potential development of the field. Digital health seems to be a general term related to information and communication technology in health care. Putting a public health lens on this general descriptive term can be done by simply expanding it towards public health as a population science and practice field, rather than the narrow medical and health care arena. However, a more specific approach towards outlining similarities and differences will also focus on digital technologies and their challenges in the core areas of prevention and health promotion. Considering the leading public health functions, their relationship with digitalisation and their specific requirements towards digitalisation can be a valuable path to describe and discuss what digital public health is all about. We will also highlight where interfaces and interrelations with digital health need to be considered for research and practice. This contribution will aim to provide such a perspective.

